# Perspective recommendations on geriatric support for older adults with multiple myeloma based on challenges faced by a multidisciplinary team

**DOI:** 10.3389/fonc.2026.1632275

**Published:** 2026-03-06

**Authors:** Julie Côté, Ploa Desforges, Christine Dionne, Frédéric Larose, Erika Martinez, Sabrina Trudel, Drena Walker

**Affiliations:** 1 Department of Hematology, Centre Hospitalier Universitaire (CHU) de Québec – Université Laval, Quebec, QC, Canada; 2 Department of Medicine, McGill University, Montreal, QC, Canada; 3 Department of Medicine, Centre Intégré Universitaire de Santé et de Services sociaux (CIUSSS) de la Capitale-Nationale, QC, Canada; 4 Department of Medicine, Université de Laval, Laval, QC, Canada; 5 Direction de soins infirmiers, Centre Intégré Universitaire de Santé et de Services Sociaux (CIUSSS) du Centre-Ouest-de l’Île-de Montréal, Montréal, QC, Canada; 6 Department of Hematology, Hôpital Charles-Le Moyne, Greenfield Park, QC, Canada; 7 Hematology Pivot Nurse Cedars Cancer Center, McGill University Health Centre and RI-MUHC, Montreal, QC, Canada

**Keywords:** adverse events, bispecific antibodies, CAR T-cells, geriatric patients, multiple myeloma, referral pathway, T-cell–redirecting therapy

## Abstract

**Background:**

Multiple Myeloma (MM) predominantly affects older adults, with a median diagnosis age of 70 years, posing challenges in management due to frailty, comorbidities, and age-related physiological changes. As the aging population grows, the prevalence of MM in older patients is expected to rise, requiring improved clinical strategies.

**Methods:**

This consensus report outlines a multidisciplinary framework for the identification, geriatric assessment (GA), and management of older adults with MM, emphasizing the role of frailty assessments, such as the IMWG frailty score, in tailoring treatments. The objective is to develop a working model tailored to the Quebec context. A meeting on May 7, 2024 brought together experts to discuss improving access to GA and developing strategies for implementing geriatric management tools. Unmet needs include the lack of standardized screening tools, limited access to specialized geriatric oncology services, and inconsistent treatment approaches. Personalized care is critical in addressing frailty, comorbidities, patient preference, and functional status.

**Results:**

This report proposes a structured referral pathway involving age-based triaging and criteria for geriatric consultation. Multidisciplinary teams, including geriatricians, are essential for optimizing care and improving outcomes, with a focus on quality of life and effective therapies. Standardized practices and collaborative approaches are vital for addressing the complexities of MM in this vulnerable population.

## Introduction

Multiple Myeloma (MM) is a hematologic malignancy characterized by the proliferation of clonal plasma cells in the bone marrow, leading to various complications. MM predominantly affects older adults, with a median age at diagnosis of approximately 70 years, and about one-third of patients are aged over 75 years (1). The projected estimate of new data for MM for 2024 is 4100 new cases in Canada and the age-standardized incidence rate is 8.4 per 100 000 (excluding Quebec) (2). According to the Quebec cancer registry, in 2022, there were 899 new cases of multiple myeloma, with 39.6% of cases occurring in patients over the age of 75 (3). Given the aging population, the prevalence of older patients with MM is expected to rise, posing significant challenges in clinical management and treatment outcomes.

Older patients with MM present unique challenges due to frailty, comorbidities, and age-related physiological changes, which complicate treatment choices and outcomes. Comorbidities such as cardiovascular diseases, diabetes, pre-existing neuropathy, and renal dysfunction further exacerbate the complexity of managing MM in this population. Consequently, these factors necessitate a careful and individualized approach to treatment to balance efficacy and tolerability (1, 4) The management of MM-related manifestations and complications focuses on the prevention, early detection, and prompt treatment of disease-related symptoms—such as bone disease, renal insufficiency, infections, and cytopenia—to improve patient quality of life while minimizing the risk of hospitalizations along with long-term sequelae and balancing treatment-related side effects. Frailty, a state of increased vulnerability resulting from age-associated declines in reserve and function across multiple physiological systems, exacerbates the complexity in MM management (5).

The standard of care for treating MM involves a combination of therapies tailored to the patient’s overall health, treatment history, and disease status (6). For older adults, particularly those who are frail, treatment regimens must be adjusted to minimize adverse events and toxicities. Standard treatments include anti-CD38 monoclonal antibodies, proteasome inhibitors (PIs) or immunomodulator drug (IMID)-based combinations. However, these treatments have limitations, especially in older and frail patients. Adverse events such as infections, peripheral neuropathy and hematologic toxicities, are more pronounced in older patients, often leading to dose modifications or discontinuation of therapy (6, 7). Moreover, ongoing trials (e.g., NCT04635189) are evaluating steroid sparing combinations with monoclonal antibodies or the role of frailty-guided treatment of older patients who may be at greater risk of treatment toxicity and poor survival (8, 9).

Additionally, the lack of personalization in treatment regimens and the underrepresentation of elderly patients in clinical trials hinder the optimization of therapeutic strategies for this demographic (10). Most clinical trials for MM have historically included younger, fitter patients, thereby limiting the generalizability of the findings to older, frail populations (10).

The Canadian Myeloma Research Group (CMRG) Consensus Guideline recommends frailty assessment for all older adults prior to treatment decision-making for transplant ineligible newly diagnosed multiple myeloma (TI NDMM). For first-line treatment, an anti-CD38-based regimen is preferred based on frailty status (6, 11). Current assessment tools for frailty recommended by oncology associations include the Karnofsky Performance Status (KPS) and Eastern Cooperative Oncology Group Performance Status (ECOG PS). While both tools consider patient age, they are oversimplified, cannot predict outcomes in the elderly, and fail to indicate functional impairment in elderly patients with cancer (6, 12, 13). The ECOG PS is based on performance status assessed by physicians, however it is prone to bias, and does not take into consideration the patient’s measurement of frailty (6, 14, 15).

Recent advancements in monoclonal antibody therapies for MM have demonstrated significant promise in patients ineligible for transplant in the first line setting. Trials such as IMROZ (NCT03319667) (16), CEPHEUS (NCT03652064) (17), BENEFIT (NCT04751877) (18), and MAIA (NCT02252172) (7) have evaluated anti-CD38-based quadruplet or triplet regimens to optimize treatment outcomes. Significant improvements were seen in progression-free survival (PFS), minimal residual disease (MRD) endpoints, achieving complete response or better. Collectively, these trials underscore the potential of anti-CD38-based triplet, and nowadays quadruplet, therapies in enhancing treatment efficacy and improving clinical outcomes in transplant-ineligible MM patients (16–19).

In the relapse setting, advanced therapies, including T-cell redirecting therapies—Chimeric antigen receptor (CAR) T-cell and bispecific antibody therapies—and, antibody-drug conjugates, are revolutionizing MM treatment by offering promising responses. CAR T-cell therapies are known for their high response rates and potential to induce complete responses in some patients, though they come with unique side effects such as cytokine release syndrome (CRS) and neurotoxicity (11). CAR-T has been evaluated in patients between 65 and 74 years of age versus 75 years of age and up. No statistically significant difference was seen between the two age groups for rates of CRS or Immune Effector Cell-Associated Neurotoxicity Syndrome (ICANS). Favorable OS and toxicity outcomes were seen in both groups indicating CAR-T use was not contraindicated in elderly patients (20). When frailty was introduced, however, efficacy was reduced in elderly frail patients treated with CAR-T, with a higher incidence of ICANS (21).

Teclistamab, a bispecific therapy, is approved in Canada for relapsed or refractory multiple myeloma patients who have received at least four prior lines of therapy (22). When evaluated in high-risk elderly patients (75 years of age and older) with RRMM, teclistamab resulted in similar response rates to the other age groups, however subgroups with other features experienced lower efficacy (23). Elranatamab is a humanized B-cell maturation antigen-CD3 bispecific antibody. Response rates were consistent across subgroups regardless of age, and have been shown to result in a similar safety profile and efficacy in elderly patients with or without frailty (24, 25). Belantamab mafodotin (Belamaf), a BCMA-targeted antibody drug conjugate, is used in combination for relapsed or refractory multiple myeloma. Used in combination with bortezomib and dexamethasone (BVd), and with pomalidomide and dexamethasone (BPd), results in ocular adverse events (AEs), which is important to consider when treating older patients (26, 27).

In addition to frailty, cognitive impairment in older patients, poses significant challenges, particularly with T-cell redirecting therapies which can cause neurological toxicities (28). Age-related cognitive declines, compounded by neurotoxicity-related AEs, can hinder treatment adherence, informed decision-making, and overall quality of life. Therefore, careful cognitive assessment before and during therapy is essential, alongside strategies like dose adjustments or alternative regimens to mitigate neurotoxic effects (29).

Managing toxicities in older patients is essential for balancing efficacy and tolerability. In older adults with multiple myeloma, frailty is often assessed using specific scoring systems that evaluate domains such as physical function, comorbidities, and cognitive status. Despite these efforts, outcomes remain suboptimal in patients aged 75 years and older, who experience a higher incidence of toxicity-related morbidity, mortality, and treatment discontinuation (30). This is especially true for transplant-ineligible patients, where toxicity-related deaths among those aged 80 years and above reach approximately 10% (30). These challenges, combined with the underrepresentation of older adults in clinical trials, highlight the critical need for early and accurate evaluation to stratify patients effectively and optimize treatment plans (30).

The involvement of geriatricians and geriatric oncologists in the management of older adults with MM is crucial (31, 32). Given that approximately one-third of patients are aged over 75 years at diagnosis (1), advanced age is a primary consideration for a geriatric assessment to address the complexities associated with aging (33, 34).

Medical societies and professional organizations emphasize the critical role of GAs and education in optimizing care for older adults with cancer, particularly those with frailty. Medical societies such as American Society of Clinical Oncology (ASCO) (34), International Society of Geriatric Oncology (SIOG) (35), National Comprehensive Cancer Network (NCCN) and Canadian Network on Aging and Cancer (CNAC) advocate for using GAs to identify vulnerabilities, stratify patients by fitness levels, and tailor treatment decisions to improve outcomes. Similarly, ASCO and SIOG provide educational resources and guidelines to equip hematologists and oncologists with the skills needed to address the complex needs of elderly patients, fostering timely and appropriate referrals to geriatric oncology care.

This consensus report aims to review the challenges in managing older adults with MM, focusing on the impact of frailty, the limitations of current treatment strategies, and the importance of integrating geriatric assessments. Here, we outline referral criteria and propose a framework for multidisciplinary care adapted towards the current Quebec healthcare system. This work seeks to provide actionable insights for optimizing outcomes in this vulnerable population.

## Objectives

Aim to develop a multidisciplinary framework for the identification, geriatric assessment and management of older adults with MM. This framework is adapted towards the current Quebec healthcare system and local realities uncovered by a meeting of experts.

## Method

On May 7, 2024, a meeting was convened bringing together seven experts (geriatricians, hemato-oncologists, nurse clinicians) with the aim of establishing a framework for the care of older adults with MM. The discussions focused on identifying pathways to enhance accessibility to geriatric assessment and management and on developing multidisciplinary strategies for the implementation of geriatric management tools. On October 28, 2024, the group met again to review tables and figures proposed at the previous meeting, propose revisions and to establish a consensus. The live meetings were supplemented with a literature review analyzing key data to guide the group’s consensus. The method for obtaining group consensus was based on a modified version of the nominal group technique (36). Patients of age 75 and older were of focus based on the results of the literature search and expert opinion.

## Results

### Key unmet needs to identify, refer, and follow up geriatric MM patients

The critical unmet needs in the management of geriatric patients with MM, emphasizes the challenges in identification, referral, and follow-up care (**Table 1**). As identified by the steering committee, these challenges highlight the necessity for standardized practices and multidisciplinary approaches to improve patient outcomes.

**Table 1:** Unmet need in MM patients.

S.No	Process	Unmet needs
1	Patient Identification	• Lack of standardized frailty assessment tools • Inadequate early detection of cognitive impairment • Limited access to comprehensive geriatric assessments
2	Referral	• Inconsistent referral practices among hematologists/oncologists • Limited awareness of the role of geriatric oncology
3	Follow Up	• Lack of standardized follow-up protocols • Limited involvement of multidisciplinary teams • Insufficient support for managing comorbidities and cognitive decline

#### Lack of screening tools for elderly patients with MM

The absence of effective and standardized screening tools for geriatric patients with MM represents a significant gap in care. Identifying frail and older patients who would benefit from specialized interventions is challenging due to inconsistent use of validated frailty and cognitive assessment methods across healthcare settings. The current frailty assessment tools such as the G8 are not standardized in clinical practice due to lack of awareness, lack of time and coordination for administering the screening tool to patients (37). As a result, critical vulnerabilities, such as physical and cognitive decline, are often overlooked. Without structured tools, healthcare providers are unable to systematically assess patients’ fitness for treatment, resulting in delayed or suboptimal disease management strategies. Employing an assortment of screening tools to gather more information will promote tailored treatments that meet the specific patient needs.

#### Challenges with toxicity profiles of MM treatments on elderly patients

The management of older adults with MM is uniquely complex due to age-associated physiological changes and the presence of multiple comorbidities (38). Patients who are frail often have reduced organ reserve, which makes them more susceptible to adverse effects from both standard and novel therapies. Moreover, many of these therapies may lead to cognitive decline which can hinder proper assessment and subsequent treatment of patients (28, 29, 39). The unique toxicity profiles of newer therapies thus require close monitoring and management from multidisciplinary teams (28, 29, 39).

Therapies must also be tailored to an individual’s ability to tolerate specific toxicities, acknowledging that newer therapies may not necessarily be less toxic than older treatments, but may offer a different toxicity profile. For example, while newer drugs may spare certain organs, they may introduce other challenges, such as neurotoxicity or increased infection incidence. This necessitates a personalized approach at the time of treatment decision, considering each patient’s frailty level, comorbidities, goals, and functional status.

#### Lack of access to specialized services

Access to specialized geriatric oncology services remains limited for many patients. In some regions, the lack of collaboration between hematologists, oncologists, and geriatricians creates barriers to comprehensive care. Local community service centers (CLSCs), which are integral to providing supportive care in Quebec, often face resource constraints, reducing their ability to assist geriatric patients. The scarcity of dedicated geriatric-oncology healthcare providers further exacerbates these challenges, leading to delays in referrals and treatment (40). Referrals to geriatric oncologists are also limited by the lack of standardized screening tools.

Patients in remote or underserved areas face additional difficulties accessing essential services, including geriatric evaluations, psychosocial support, and nutritional counseling. These gaps highlight the urgent need for systemic changes to improve the availability and accessibility of geriatric oncology resources.

#### Need for standardized treatments and tools within clinical practice

There is also a pressing need to establish standardized treatment approaches across centers, ensuring that therapies are adjusted based on frailty assessments. Proactive geriatric assessment (GA) can guide therapy selection, balancing efficacy and toxicity while considering the patient’s individual needs and goals of care. For instance, some patients may benefit from dose-reduced regimens to minimize toxicity without compromising efficacy (40).

The management of geriatric patients with MM necessitates the use of standardized tools for assessment across all treatment centers. Implementing consistent GA practices would enable healthcare providers to monitor patient progress effectively, guide treatment decisions, and facilitate comparisons of outcomes across institutions. Such tools could help identify frailty, cognitive decline, and other geriatric-specific vulnerabilities, promoting timely and appropriate interventions. While standardized tools for frailty assessment provide valuable frameworks, however, their limited integration into routine practice and inconsistency in how the frailty assessment is interpreted remain a significant barrier in delivering optimal care to this population (12, 41).

#### Communication gaps between disciplines

A critical need exists to improve communication between hematologists and geriatricians, ensuring a collaborative approach to care. Proactive assessments, conducted upon diagnosis and before the start of first and subsequent line therapy, are essential to guide treatment plans tailored to the unique needs of older adults. This approach is far more effective than the current reactive model, which often delays interventions. Additionally, the question of responsibility for screening is a concern. Hematologists are skilled in oncology-specific evaluations, but they may not be equipped with the tools or training to assess multidimensional aspects of frailty, such as cognitive function, nutrition, and psychosocial factors. Moreover, hematologists often face high patient loads and time constraints, limiting their ability to perform detailed evaluations or coordinate with geriatricians effectively. Promoting communication within the multiple disciplines involved in care would alleviate the burdens placed on both patients and HCPs. We recommend an organized and standardized referral pathway clearly indicating the roles of each discipline to prevent miscommunication and delays in screening and intervention.

#### Political action is needed

A political commitment is also essential to drive systemic change. Currently, there is a lack of institutional guidelines mandating routine screenings at every treatment center. Organizations like the Quebec Cancer Program (QCP) must advocate for and establish guidelines to ensure standardized screening practices are universally adopted.

#### Need for consistency amongst clinical care

The frequency and timing of assessments are equally crucial. Regular evaluations can help monitor patient responses to therapy, enabling adjustments based on changes in patient factors or dosing requirements. Simple follow-up questions about weight loss, falls, or other functional declines can provide valuable insights, especially when collected systematically by a multidisciplinary team (42).

An additional challenge is the inconsistency in medical records across hospitals, with some still relying on paper-based systems while others use electronic health records. Critical patient data, such as weight, is often missing or difficult to find from these records, complicating the evaluation process. A systematic approach to capturing and monitoring elements such as patient weight, integrated into the medical record system, is crucial.

#### Need for multidisciplinary involvement in care

Geriatricians play a vital role in ensuring treatment feasibility for patients who are frail, particularly those with cognitive impairment, by offering strategies to mitigate toxicities and support overall care. Standardized tools, multidisciplinary approaches, and improved resource allocation are critical to overcoming these challenges and improving outcomes for geriatric patients with MM.

Additionally, the lack of dedicated geriatric oncology healthcare professionals (HCPs) in some healthcare centers exacerbates this issue (38). Geriatric oncologists and geriatricians specialized in oncology play a critical role in assessing frailty, cognitive function, and comorbidities that influence treatment decisions for older adults with MM. In centers without these professionals, oncologists often lack the specialized training required to conduct GAs, creating a gap in care delivery.

A cultural shift in hematology is needed to prioritize the assessment of elderly patients early in the treatment process. Early GA helps identify patients’ unique vulnerabilities, such as frailty, polypharmacy, or social challenges, allowing for more personalized and effective treatment plans. General practitioners (GPs) could play a pivotal role in this process by conducting preliminary assessments, including cognitive testing, before referring patients to geriatric oncology services. However, this approach is often impractical because many elderly patients do not have consistent access to family doctors (40). The lack of primary care continuity further limits the effectiveness of GPs in facilitating referrals.

Geriatricians also consider the availability of social support or caregivers in their evaluations to ensure that patients, particularly those with cognitive impairments, have the necessary support system for attending appointments and managing medications and eventual side effects of treatment. This holistic approach ensures that the treatment plan accounts not only for the medical needs of the patient but also for the logistical and social aspects that may influence adherence to care.

Finally, HCPs need to take a proactive role in educating patients’ families and caregivers about the role of geriatricians in the treatment process (33). By increasing awareness, caregivers can better understand the importance of geriatric input, enabling them to support patients more effectively. Education initiatives can include explaining how geriatricians address treatment feasibility, manage toxicities, and support patients’ overall well-being.

Older adults with MM experience additional hurdles compared to the average patient with MM. By identifying the challenges that some of the most vulnerable of patients with MM, we uncover weaknesses within the healthcare system. Addressing these challenges requires a multifaceted approach, including expanding access to specialized services, training additional geriatric oncologists, involving GPs in preliminary evaluations where feasible, educating hematology and oncology nurses of specific needs of older adults with cancer, and enhancing caregiver education to optimize the referral and treatment process. This emphasizes the benefits of a multidisciplinary approach to be incorporated into an updated referral pathway.

### Essential criteria and characteristics for identifying patients who should be referred to geriatric oncology

The identification of older adults with MM who would benefit from a referral to geriatric oncology requires careful evaluation of key factors, tools, and challenges (**Table 2**). GA serves as a cornerstone for this process, enabling healthcare providers to assess frailty, comorbidities, and other vulnerabilities that may affect treatment outcomes. Below are the essential criteria and characteristics for referral, as well as a discussion on the tools and methods used for frailty detection, along with their advantages and limitations.

**Table 2:** Characteristics used for the identification of patients who would benefit from referral to geriatric oncology.

S.No	Factors	Examples
1	Age	Patients >85 years; those showing signs of frailty or cognitive decline
2	Comorbidities	Cardiovascular diseases, chronic pulmonary diseases, chronic kidney diseases, diabetes, malnutrition
3	Cognitive Impairment	Memory loss, difficulty in decision making, dementia
4	Access to care	Geographic location, availability of specialized geriatric oncology services and CLSC, transportation difficulties
5	Functional Status	Decreased mobility, difficulty in performing daily activities, problems with visual acuity or hearing acuity
6	Psychosocial factors	Lack of family support, depression, anxiety
7	Cancer-related symptoms	Persistent pain, unexplained weight loss
8	Treatment history	Previous cancer treatments, history of adverse reactions, resistance to standard therapies
9	Patient preferences	Desire for aggressive treatment, preference for quality of life over life extension
10	Healthcare system factors	Availability of multidisciplinary teams, time constraints on HCPs, length/complexity of referral process

Factors: a list of key aspects to consider when identifying geriatric oncology patients. Examples: for each factor to illustrate what HCPs should look for..

CLSC, centre local de services communautaires (local community services center); HCP, healthcare professional; MM, multiple myeloma..

Key factors for patient identification for referral include age, cognitive abilities, frailty, and the presence of significant comorbidities. A specific threshold for these criteria needs to be established in clinical settings to ensure consistent and effective patient identification. For example, older adults with measurable frailty or cognitive impairments, as well as those exhibiting continuous clinical deterioration or lack of improvement despite treatment, should be prioritized for referral to geriatric oncology (1, 30).

Comorbidities play a significant role in influencing treatment decisions. (5, 41). In addition to managing comorbidities, MM presents a wide range of disease manifestations and complications, which are exacerbated by the patient’s advanced age and significantly impair quality of life. These complications require targeted management strategies to mitigate their impact (5). The CMRG Consensus Guideline Consortium emphasizes the need for a national consensus on the prevention, detection, and management of MM-related manifestations and complications. These guidelines provide evidence-based recommendations that consider the older median age of patients with MM and are aimed at improving and maintaining patient care across Canada (5).

The IMWG frailty score is an additive scoring system (0–5 points) that combines geriatric evaluation (functional status in activities of daily living (ADL), instrumental activities of daily living(IADL)), and age to classify patients into three groups: “fit” (score = 0), “intermediate fitness” (score = 1), and “frail” (score ≥2), with a score ≥2 being considered the gold standard to signify frailty (15, 30, 41). On the other hand, simplified myeloma frailty scale is designed to categorize patients based on fewer parameters, enhancing its feasibility using only two categories: nonfrail (score = 0–1) and frail (score ≥2) (14). The IMWG and simplified myeloma frailty scores are MM-specific (14, 41). Complementing this, CGA is a multidimensional, evidence-based tool that evaluates social status, comorbidities, functional and mental states, polypharmacy, nutritional status, and geriatric syndromes. This approach identifies deficits that may not be detected through routine clinical examinations, guiding treatment decisions in older oncology patients. While rarely performed, CGA has been shown to be successfully used in patients with MM (43). However, GA is preferred, carried out without the multidisciplinary team (13).

Frailty assessment tools offer significant advantages in managing older patients with MM, particularly in tailoring treatment approaches. By integrating factors such as age, functional status, and comorbidities, these tools effectively predict survival rates and treatment-related toxicity (12). GA facilitate treatment personalization, minimizing toxicity and improving clinical outcomes. Moreover, GA implementation has been shown to reduce the risk of severe AEs by approximately one-third, enhancing patient safety during treatment (12). These assessments also improve therapeutic decision-making, ensuring that frail patients receive care aligned with their functional and clinical needs. Additionally, including frail patients in clinical trials generates more applicable data for this vulnerable population, contributing to the broader relevance of research findings.

However, frailty assessments are not without challenges (44). The time-intensive and resource-demanding nature of these evaluations further hampers their routine use in clinical practice. Additionally, standard performance status tools, such as Eastern Cooperative Oncology Group (ECOG) scores, often fall short, necessitating more advanced methods for comprehensive frailty evaluation.

Post-diagnosis follow-up is crucial for the ongoing management of a patient’s health conditions, ensuring continuous care for chronic or comorbid issues alongside their treatment journey. Moreover, MM treatment is typically continuous as most patients will relapse and undergo several lines of treatment throughout their journey. This necessitates the ongoing evaluation of frailty due to its dynamic nature. Due to the dynamic state of increased vulnerability to stressors, frailty is not static or inevitable but can evolve over time. The dynamic nature of frailty highlights its potential for progression, stability, or improvement, depending on interventions and health management strategies (42, 45). As frailty changes over time, it also affects treatment options, tolerance, and overall patient outcomes (43, 46).

Geriatricians play a vital role in monitoring patients after referral, often collaborating with oncologists to deliver integrated care (47). GA contributes to developing personalized treatment plans that address individual needs, enhancing the overall quality of care and improving outcomes for elderly patients with complex health conditions (41).

### Referral framework to geriatric oncology for patients with MM

**Figure 1:**
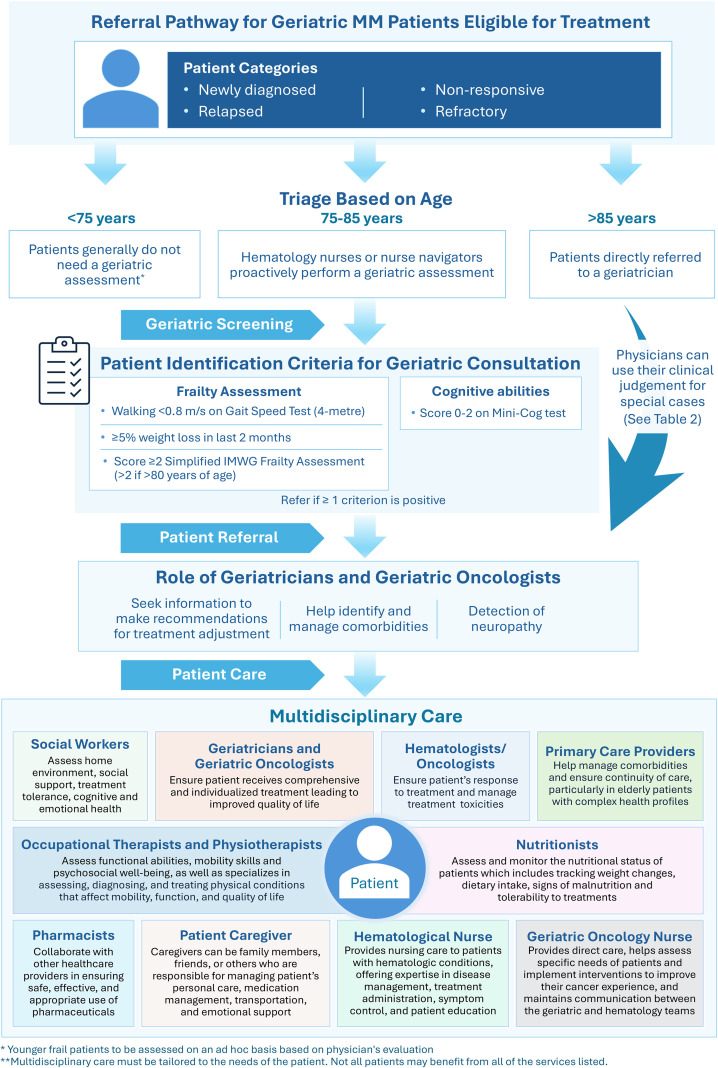
Referral pathway for geriatric MM patients eligible for treatment. Multidisciplinary care must be tailored to the complex needs of the patient in a way that optimizes quality of life and treatment outcome. Not all patients may benefit from every service listed in the algorithm.

As a solution to the aforementioned challenges, we propose a pathway designed to ensure that geriatric patients with MM receive appropriate, individualized assessments and interventions, according to the challenges unveiled in the Quebec healthcare system. This structured approach we have developed (**Figure 1**) helps identify patients who may benefit from adjustment of treatment or from geriatric consultation, ensuring that their complex needs are managed in a way that optimizes their quality of life and treatment outcomes. The pathway begins with age-based patient triage, segmenting patients by age group to determine the level of GA required:

- Under 75 Years: Patients younger than 75 typically do not need to undergo GA as they are presumed to have fewer age-related health issues (13). However, GA should still be considered based on the clinical judgment of HCPs, especially if the patient has comorbidities, functional decline, or other factors that could impact treatment decisions.
- Ages 75-85: Patients in this age range should undergo an initial geriatric screening conducted by a hematologist or hematology nurses or nurse navigators. This proactive approach helps identify those who may benefit from further geriatric consultation based on frailty or cognitive decline.
- Over 85 Years: Patients over 85 for whom a treatment is considered should be directly referred to a geriatrician due to the likelihood of age-related health complexities, making a comprehensive geriatric evaluation essential.

For patients in the 75–85 age group, specific criteria are used to guide the decision for referral to a geriatric consultation. This assessment includes— Geriatric screening: Indicators like gait speed (less than 0.8 m/s), recent weight loss (5% or more in the last two months), and a Simplified IMWG Frailty Score (≥2, or, if the patient is over 80 years old, >2) help determine physical frailty levels. Cognitive Assessment: Cognitive ability is evaluated using the Mini-Cog test, selected based on expert opinion, with a score of 0–2 suggesting potential cognitive challenges (48). The chosen assessments provide a plethora of information on the functional and cognitive status of the patient more so than the standard tools currently used, allowing clinicians to identify patients who may benefit from a comprehensive geriatric assessment and support. While the criteria are structured, physicians retain the flexibility to use clinical judgment in special cases such as patients with acute medical conditions that temporarily affect cognitive function, or those with exceptional resilience despite advanced age (**Figure 1**). This allows them to make tailored decisions for patients who may not meet the exact criteria but still require geriatric consultation.

Once a referral is made using on the proposed screening of patient characteristics, geriatricians and geriatric oncologists conduct a thorough geriatric assessment, evaluating domains such as physical function, cognitive status, nutrition, and social support (**Table 2**). This comprehensive assessment informs personalized geriatric management strategies, ensuring that care plans are tailored to address the patient’s specific life goals, needs and vulnerabilities. Their responsibilities would include making treatment recommendations based on the patient’s overall health and age-related conditions. The geriatric oncology team will also put interventions in place to optimize functioning and psychosocial support to facilitate treatment experience. They advise on adjusting MM treatment regimens to improve safety and effectiveness. Comorbidity management where, geriatricians help identify and manage comorbid conditions that may interfere with MM treatment or exacerbate health risks and neuropathy detection. As an example, neuropathy is a common side effect of MM treatments, geriatricians monitor and address this condition, especially for patients who are more susceptible to treatment-related complications.

Steroid tapering in elderly MM patients requires individualized strategies to balance efficacy and minimize toxicity and highlights the roles that various healthcare professionals play in the effective management of elderly MM patients (8). Alternatively, steroid-free approaches have been successfully implemented in elderly patients with MM and should be considered (49).

### Follow-up and multidisciplinary care

Geriatricians and geriatric oncologists play vital roles in frailty assessment, treatment personalization, and managing toxicities, including steroid-related side effects. Their multidisciplinary approach ensures optimized care, focusing on quality of life and effective therapy, while fostering coordination among care teams to address the unique needs of this vulnerable patient population. Following the geriatric consultation, patients may benefit from a multidisciplinary care approach (**Figure 1**), where various specialists collaborate to provide holistic and comprehensive support. It is important to note that access to multidisciplinary care may vary across clinics. Social workers can assess the patient’s home environment, identify available resources, and provide guidance on accessing community or institutional support systems to address psychosocial and practical challenges. They also assist in coordinating care plans and ensuring patients and caregivers have the tools needed to navigate their treatment journey. Occupational therapists may be asked to evaluate the patient’s functional abilities, mobility, and overall well-being, working to maintain or improve their independence and quality of life. Pharmacists collaborate with other healthcare providers to ensure the safe, effective, and appropriate use of medications, carefully managing polypharmacy by addressing potential interactions, side effects, and the overall medication burden on patients. Hematologists oversee MM-specific treatment, monitoring the patient’s response to therapy and managing treatment-related toxicities, especially for patients with age-related vulnerabilities. Nutritionists track dietary intake, nutritional status, and any signs of malnutrition or treatment intolerance, offering tailored nutritional plans to support the patient’s overall health. Hematological nurses provide direct care, focusing on disease management, symptom control, and patient education, helping patients effectively manage MM and related symptoms. Geriatric oncology nurses also provide direct care. They help assess specific needs for older adults and implement interventions to improve their cancer experience. Geriatric oncology nurses also aid in maintaining communication between the geriatric and hematology teams. Through this coordinated, patient-centered approach, the multidisciplinary team aims to enhance the patient’s quality of life and treatment outcomes.

## Discussion

Based on insights from a multidisciplinary meeting of HCPs specializing in the management of older adults with multiple myeloma, we propose a referral algorithm designed to streamline and optimize care for this vulnerable population. While this algorithm addresses several critical gaps in the care pathway, some limitations must be acknowledged to further refine and enhance its utility.

### Limitations of the algorithm

The proposed algorithm identifies key factors influencing patient referral and follow-up in geriatric MM care. However, certain components require further elaboration such as:

- Patient Follow-Up and Reassessment Plan: The algorithm lacks detailed guidance on follow-up intervals and reassessment strategies. Regular monitoring is essential to capture changes in physical and cognitive health, functional status, and treatment tolerance. Clear recommendations on how often and by whom reassessments should be conducted would enhance its clinical applicability.
- Involvement of Primary Care Providers: While the algorithm incorporates a multidisciplinary approach, it does not provide explicit instructions on integrating primary care providers. Primary care providers are pivotal in managing comorbidities and ensuring continuity of care, particularly in elderly patients with complex health profiles.
- Applicability of the findings: Based on discussions with the HCPs from Québec, the results and conclusions of the meetings may not be generalizable to all of Canada. While the insights can inform practices in other regions, healthcare systems, resources, and referral pathways may vary significantly across provinces and territories, affecting the applicability of the proposed framework.

In addition, the algorithm refers to an ideal scenario, where multiple disciplines are available. While multidisciplinary care should be standardized, there are socio-economic and local disparities that may hinder access to care and resources.

### Scope of the algorithm

A critical feature of the algorithm is its emphasis on conducting baseline assessments for all patients with MM. Establishing a comprehensive baseline assessment at diagnosis provides a reference point for evaluating changes in health over time.

This approach offers multiple benefits such as:

- Facilitates Early Identification of Changes: A standardized baseline enables clinicians to promptly identify shifts in physical, cognitive, and functional status, ensuring timely referrals and interventions.
- Supports Multidisciplinary Collaboration: Baseline data serves as a shared foundation for social workers, occupational therapists, pharmacists, hematologists, and other team members. This ensures a cohesive and informed approach to care planning.
- Tailors Treatment Plans: Baseline knowledge allows treatment to be customized to the patient’s unique needs, maximizing efficacy while minimizing risks.

Addressing Unmet Needs

The algorithm addresses several longstanding unmet needs in geriatric MM care by providing a structured and individualized framework for patient management. The key strengths of the algorithm include:

- Consideration of Physical Frailty and Cognitive Function: Proactive assessment ensures that the pathway accommodates the specific vulnerabilities of older patients, enabling more precise and effective treatment strategies.
- Emphasis on Social Support: By integrating the role of caregivers and social networks, the pathway acknowledges the broader context of patient well-being, which is often overlooked in traditional care models.
- Integration of Nutritional Support: Older adults with MM often experience weight loss and malnutrition during treatment, which negatively impacts their overall health and ability to tolerate therapy. However, systematic nutritional screening and timely referrals to dietitians are rarely integrated into standard care protocols. Regular weight monitoring and tailored nutritional interventions should be implemented to support these patients throughout their treatment journey.
- Comprehensive, Patient-Centered Care: By incorporating diverse medical and social factors, the pathway shifts the focus from disease-centered to patient-centered care. This approach aims to improve clinical outcomes while enhancing the quality of life for geriatric patients with MM.

Embedding GA into oncology workflows ensures seamless integration and accessibility for elderly patients, minimizing disruption to existing processes. Training programs for nurses and physicians equip HCPs with the skills to conduct initial screenings, identify frailty, and refer patients effectively. Standardized tools, such as the Mini-Cog and gait speed tests, bring consistency across practices, ensuring uniformity in assessments. This structured approach not only facilitates the early identification of high-risk patients but also streamlines the coordination between oncology and geriatric services.

The implementation of this algorithm (**Figure 1**) is expected to yield benefits. By tailoring treatment intensity for old and frail patients, adverse effects can be minimized, improving treatment tolerance and overall quality of life. Comprehensive care plans address broader health needs, enhancing functional status and social support. Moreover, targeted referrals optimize resource allocation, preventing overburdening of geriatric services while improving survival outcomes through balanced, personalized interventions that prioritize patient well-being.

### Future directions

Although the algorithm represents a significant advancement in geriatric MM care, further research and clinical validation are necessary to refine its components. Addressing its limitations, particularly in follow-up and multidisciplinary integration, will be essential for ensuring its widespread adoption and effectiveness.

We hope these recommendations provide clarity and actionable insights for HCPs in managing older adults with MM. By fostering a more cohesive and personalized approach to care, this framework aims to set a new standard for the management of MM in aging populations.

### Limitations of the consensus statement

Given that the therapeutic landscape of MM is rapidly evolving and that geriatric MM care is an area of intensive research, we have presented recommendations based on the most up-to-date evidence and clinical experience at the time of writing. We expect best practices to advance as real-world experience with geriatric MM care accumulates and as clinical data mature.

## Conclusions

Here, we have presented a set of perspective recommendations for optimizing geriatric support for older adults with multiple myeloma from Quebec hematologists, nurses and geriatricians. This set of recommendations, which is expected to evolve in the future, includes a proposed algorithm for implementing a proactive geriatric assessment, coordination of care, patient selection, clinical decision-making, and adverse event management. Implementation of these protocols is expected to streamline health care resource utilization, to improve geriatric support for older adult patients with multiple myeloma and help identify frailty in these individuals.

## Data Availability

The original contributions presented in the study are included in the article/supplementary material. Further inquiries can be directed to the corresponding author/s.

## References

[1] Facon T Leleu X Manier S . How I treat multiple myeloma in geriatric patients. Blood. (2023) 143:224–32. doi: 10.1182/blood.2022017635, PMID: 36693134 PMC10808246

[2] Brenner D Gillis J Demers AA Ellison LF Billette JM Zhang SX . Projected estimates of cancer in Canada in 2024. CMAJ. (2024) 196:E615–23. doi: 10.1503/cmaj.240095, PMID: 38740416 PMC11090635

[3] Quebec Cancer Registry . Statistiques du Registre Québécois du Cancer (2025). Available online at: https://app.powerbi.com/view?r=eyJrIjoiNjc2ZTAxNmMtMWFiMi00NDIwLTg0MzYtOTY2OTIzMDliYjA2IiwidCI6IjA2ZTFmZTI4LTVmOGItNDA3NS1iZjZjLWFlMjRiZTFhNzk5MiJ9 (Accessed February 27, 2026).

[4] Grant SJ Freeman CL Rosko AE . Treatment of older adult or frail patients with multiple myeloma. Hematol Am Soc Hematol Educ Program. (2021) 2021:46–54. doi: 10.1182/hematology.2021000231, PMID: 34889397 PMC8791156

[5] LeBlanc R Bergstrom DJ Côté J Kotb R Louzada ML Sutherland HJ . Management of Myeloma Manifestations and Complications: The Cornerstone of Supportive Care: Recommendation of the Canadian Myeloma Research Group (formerly Myeloma Canada Research Network) Consensus Guideline Consortium. Clin Lymphoma Myeloma Leuk. (2022) 22:e41–56. doi: 10.1016/j.clml.2021.07.028, PMID: 34456159

[6] Côté J Kotb R Bergstrom DJ LeBlanc R Mian HS Othman I . First Line Treatment of Newly Diagnosed Transplant Ineligible Multiple Myeloma: Recommendations from the Canadian Myeloma Research Group Consensus Guideline Consortium. Clin Lymphoma Myeloma Leuk. (2023) 23:340–54. doi: 10.1016/j.clml.2023.01.016, PMID: 36925389

[7] Facon T Moreau P Weisel K Goldschmidt H Usmani SZ Chari A . Daratumumab/lenalidomide/dexamethasone in transplant-ineligible newly diagnosed myeloma: MAIA long-term outcomes. Leukemia. (2025) 39:942–50. doi: 10.1038/s41375-024-02505-2, PMID: 40016302 PMC11976258

[8] McKenzie F Gazzé G Hewitt J Kolm K Pollock D Rowland S . Canadian perspectives in multiple myeloma on the use of steroids in clinical practice based on patient and healthcare provider interviews. Front Oncol. (2022) 12:1061417. doi: 10.3389/fonc.2022.1061417, PMID: 36568227 PMC9772426

[9] Steroid sparing treatment with in newly diagnosed transplant ineligible patients with multiple myeloma. Available online at: https://ClinicalTrials.gov/show/NCT04635189 (Accessed February 27, 2026).

[10] Mian H Wildes TM Venner CP Fonseca R . MRD negativity: considerations for older adults with multiple myeloma. Blood Cancer J. (2023) 13:166. doi: 10.1038/s41408-023-00939-y, PMID: 37938551 PMC10632351

[11] Agency CsD . Multiple Myeloma. Available online at: https://www.cda-amc.ca/multiple-myeloma-1 (Accessed February 27, 2026).

[12] Kaweme NM Changwe GJ Zhou F . Approaches and Challenges in the Management of Multiple Myeloma in the Very Old: Future Treatment Prospects. Front Med (Lausanne). (2021) 8:3389/fmed.2021.612696. doi: 10.3389/fmed.2021.612696, PMID: 33718400 PMC7947319

[13] Wildiers H Heeren P Puts M Topinkova E Janssen-Heijnen ML Extermann M . International Society of Geriatric Oncology consensus on geriatric assessment in older patients with cancer. J Clin Oncol. (2014) 32:2595–603. doi: 10.1200/JCO.2013.54.8347, PMID: 25071125 PMC4876338

[14] Facon T Dimopoulos MA Meuleman N Belch A Mohty M Chen WM . A simplified frailty scale predicts outcomes in transplant-ineligible patients with newly diagnosed multiple myeloma treated in the FIRST (MM-020) trial. Leukemia. (2020) 34:224–33. doi: 10.1038/s41375-019-0539-0, PMID: 31427722 PMC7214253

[15] Cook G Larocca A Facon T Zweegman S Engelhardt M . Defining the vulnerable patient with myeloma-a frailty position paper of the European Myeloma Network. Leukemia. (2020) 34:2285–94. doi: 10.1038/s41375-020-0918-6, PMID: 32555295 PMC7449877

[16] Facon T Dimopoulos MA Leleu XP Beksac M Pour L Hajek R . Phase 3 study results of isatuximab, bortezomib, lenalidomide, and dexamethasone (Isa-VRd) versus VRd for transplant-ineligible patients with newly diagnosed multiple myeloma (IMROZ). J Clin Oncol. (2024) 42:7500–0. doi: 10.1200/JCO.2024.42.16_suppl.7500, PMID: 41735675

[17] Usmani SZ Facon T Hungria V Bahlis NJ Venner CP Braunstein M . Daratumumab plus bortezomib, lenalidomide and dexamethasone for transplant-ineligible or transplant-deferred newly diagnosed multiple myeloma: the randomized phase 3 CEPHEUS trial. Nat Med. (2025) 31:1195–202. doi: 10.1038/s41591-024-03485-7, PMID: 39910273 PMC12003169

[18] Leleu X Hulin C Lambert J Bobin A Perrot A Karlin L . Isatuximab, lenalidomide, dexamethasone and bortezomib in transplant-ineligible multiple myeloma: the randomized phase 3 BENEFIT trial. Nat Med. (2024) 30:2235–41. doi: 10.1038/s41591-024-03050-2, PMID: 38830994 PMC11333283

[19] Usmani SZ Facon T Hungria V Bahlis NJ Venner CP Braunstein M . OA-63 Daratumumab + Bortezomib/Lenalidomide/Dexamethasone in Patients With Transplant-ineligible or Transplant-deferred Newly Diagnosed Multiple Myeloma: Results of the Phase 3 CEPHEUS Study. Clin Lymphoma Myeloma Leukemia. (2024) 24:S288–9. doi: 10.1016/S2152-2650(24)02344-9, PMID: 41698777

[20] Johnson PC Neckermann I Sadrzadeh H Newcomb R El-Jawahri AR Frigault MJ . Clinical Outcomes and Toxicity in Older Adults Receiving Chimeric Antigen Receptor T Cell Therapy. Transplant Cell Ther. (2024) 30:490–9. doi: 10.1016/j.jtct.2024.02.019, PMID: 38412928

[21] Davis JA Dima D Ahmed N DeJarnette S McGuirk J Jia X . Impact of Frailty on Outcomes after Chimeric Antigen Receptor T Cell Therapy for Patients with Relapsed/Refractory Multiple Myeloma. Transplant Cell Ther. (2024) 30:298–305. doi: 10.1016/j.jtct.2023.12.015, PMID: 38142943

[22] Myeloid Canada . Health canada approves the first and only anti-bcma car t cell therapy for multiple myeloma. Available online at: https://myeloma.ca/2021/05/31/health-canada-approves-the-first-and-only-anti-bcma-car-t-cell-therapy-for-multiple-myeloma/ (Accessed February 27, 2026).

[23] Luciano JC Nizar JB Saad ZU Niels WCJ Ajay KN Aurore P . MM-328 Efficacy and Safety of Teclistamab in Patients With Relapsed/Refractory Multiple Myeloma (RRMM) With High-Risk (HR) Features: a Subgroup Analysis From the Phase 1/2 MajesTEC-1 Study. Clin Lymphoma Myeloma Leukemia. (2024) 24:S546–7. doi: 10.1016/S2152-2650(24)01664-1

[24] Lesokhin AM Tomasson MH Arnulf B Bahlis NJ Miles Prince H Niesvizky R . Elranatamab in relapsed or refractory multiple myeloma: phase 2 MagnetisMM-3 trial results. Nat Med. (2023) 29:2259–67. doi: 10.1038/s41591-023-02528-9, PMID: 37582952 PMC10504075

[25] Leleu X Raje N Lesokhin A Mohty M Nooka A Leip E . P880: Efficacy and safety of elranatamab by age and frailty in patients with relapsed/refractory multiple myeloma (RRMM): a subgroup analysis from magnetismm-3. Hemasphere. (2023) 7:e051743c. doi: 10.1097/01.HS9.0000970424.05174.3c, PMID: 33079766

[26] Trudel S Beksac M Pour L Delimpasi S Quach H Vorobyev VI . Results from the randomized phase 3 DREAMM-8 study of belantamab mafodotin plus pomalidomide and dexamethasone (BPd) vs pomalidomide plus bortezomib and dexamethasone (PVd) in relapsed/refractory multiple myeloma (RRMM). J Clin Oncol. (2024) 42:LBA105–5. doi: 10.1200/JCO.2024.42.17_suppl.LBA105, PMID: 41735675

[27] Mateos M-V Robak P Hus M Xia Z Zherebtsova V Ward C . Results from the randomized phase III DREAMM-7 study of belantamab mafodotin (belamaf) + bortezomib, and dexamethasone (BVd) vs daratumumab, bortezomib, and dexamethasone (DVd) in relapsed/refractory multiple myeloma (RRMM). J Clin Oncol. (2024) 42:439572–2. doi: 10.1200/JCO.2024.42.36_suppl.439572, PMID: 41735675

[28] Markouli M Ullah F Unlu S Omar N Lopetegui-Lia N Duco M . Toxicity Profile of Chimeric Antigen Receptor T-Cell and Bispecific Antibody Therapies in Multiple Myeloma: Pathogenesis, Prevention and Management. Curr Oncol. (2023) 30:6330–52. doi: 10.3390/curroncol30070467, PMID: 37504327 PMC10378049

[29] Jones D Vichaya EG Wang XS Sailors MH Cleeland CS Wefel JS . Acute cognitive impairment in patients with multiple myeloma undergoing autologous hematopoietic stem cell transplant. Cancer. (2013) 119:4188–95. doi: 10.1002/cncr.28323, PMID: 24105672 PMC3834212

[30] Bonello F Boccadoro M Larocca A . Diagnostic and Therapeutic Challenges in the Management of Intermediate and Frail Elderly Multiple Myeloma Patients. Cancers (Basel). (2020) 12:3106. doi: 10.3390/cancers12113106, PMID: 33114320 PMC7690866

[31] Loh KP Liposits G Arora SP Neuendorff NR Gomes F Krok-Schoen JL . Adequate assessment yields appropriate care-the role of geriatric assessment and management in older adults with cancer: a position paper from the ESMO/SIOG Cancer in the Elderly Working Group. ESMO Open. (2024) 9:103657. doi: 10.1016/j.esmoop.2024.103657, PMID: 39232585 PMC11410714

[32] Wildes TM Campagnaro E . Management of multiple myeloma in older adults: Gaining ground with geriatric assessment. J geriatric Oncol. (2017) 8:1–7. doi: 10.1016/j.jgo.2016.04.001, PMID: 27118356 PMC5075272

[33] Mian H McCurdy A Giri S Grant S Rochwerg B Winks E . The prevalence and outcomes of frail older adults in clinical trials in multiple myeloma: A systematic review. Blood Cancer J. (2023) 13:6. doi: 10.1038/s41408-022-00779-2, PMID: 36599867 PMC9813365

[34] Williams GR Hopkins JO Klepin HD Lowenstein LM Mackenzie A Mohile SG . Practical Assessment and Management of Vulnerabilities in Older Patients Receiving Systemic Cancer Therapy: ASCO Guideline Questions and Answers. JCO Oncol Pract. (2023) 19:718–23. doi: 10.1200/OP.23.00263, PMID: 37459585

[35] Burhenn PS McCarthy AL Begue A Nightingale G Cheng K Kenis C . Geriatric assessment in daily oncology practice for nurses and allied health care professionals: Opinion paper of the Nursing and Allied Health Interest Group of the International Society of Geriatric Oncology (SIOG). J geriatric Oncol. (2016) 7:315–24. doi: 10.1016/j.jgo.2016.02.006, PMID: 26961585

[36] Harvey N Holmes CA . Nominal group technique: an effective method for obtaining group consensus. Int J Nurs Pract. (2012) 18:188–94. doi: 10.1111/j.1440-172X.2012.02017.x, PMID: 22435983

[37] De Schrevel J Faucon C Sibille FX Dumont L Herrmann FR Rouvière H . The Edmonton Frail Scale as a screening score for frailty in oncogeriatrics. Front Med (Lausanne). (2024) 11:1466366. doi: 10.3389/fmed.2024.1466366, PMID: 39403280 PMC11471611

[38] Pathi N Parikh PM Banerjee J Tilak T Prem NN Pillai A . Unmet Needs in Geriatric Oncology. South Asian J Cancer. (2023) 12:221–7. doi: 10.1055/s-0043-1771441, PMID: 37969679 PMC10635778

[39] Sonneveld P Dimopoulos MA Boccadoro M Quach H Ho PJ Beksac M . Daratumumab, Bortezomib, Lenalidomide, and Dexamethasone for Multiple Myeloma. N Engl J Med. (2024) 390:301–13. doi: 10.1056/NEJMoa2312054, PMID: 38084760

[40] Cook S Alibhai S Mehta R Savard MF Mariano C LeBlanc D . Improving Care for Older Adults with Cancer in Canada: A Call to Action. Curr Oncol. (2024) 31:3783–97. doi: 10.3390/curroncol31070279, PMID: 39057151 PMC11275828

[41] Palumbo A Bringhen S Mateos MV Larocca A Facon T Kumar SK . Geriatric assessment predicts survival and toxicities in elderly myeloma patients: an International Myeloma Working Group report. Blood. (2015) 125:2068–74. doi: 10.1182/blood-2014-12-615187, PMID: 25628469 PMC4375104

[42] Mian H Wildes TM Vij R Pianko MJ Major A Fiala MA . Dynamic frailty risk assessment among older adults with multiple myeloma: A population-based cohort study. Blood Cancer J. (2023) 13:76. doi: 10.1038/s41408-023-00843-5, PMID: 37164972 PMC10172354

[43] Scheubeck S Ihorst G Schoeller K Holler M Möller MD Reinhardt H . Comparison of the prognostic significance of 5 comorbidity scores and 12 functional tests in a prospective multiple myeloma patient cohort. Cancer. (2021) 127:3422–36. doi: 10.1002/cncr.33658, PMID: 34061991

[44] Mian H Brouwers M Kouroukis CT Wildes TM . Comparison of Frailty Scores in Newly Diagnosed Patients with Multiple Myeloma: A Review. J Frailty Aging. (2019) 8:215–21. doi: 10.14283/jfa.2019.25, PMID: 31637409 PMC12275661

[45] Abbasi M Rolfson D Khera AS Dabravolskaj J Dent E Xia L . Identification and management of frailty in the primary care setting. CMAJ. (2018) 190:E1134–40. doi: 10.1503/cmaj.171509, PMID: 30249759 PMC6157492

[46] Kastritis E Palumbo A Dimopoulos MA . Treatment of relapsed/refractory multiple myeloma. Semin Hematol. (2009) 46:143–57. doi: 10.1053/j.seminhematol.2009.01.004, PMID: 19389498

[47] Marshall EG Miller L Moritz LR . Challenges and impacts from wait times for specialist care identified by primary care providers: Results from the MAAP study cross-sectional survey. Healthc Manage Forum. (2023) 36:340–6. doi: 10.1177/08404704231182671, PMID: 37415463 PMC10448708

[48] Williams GR Hopkins JO Klepin HD Lowenstein LM Mackenzie A Mohile SG . Practical Assessment and Management of Vulnerabilities in Older Patients Receiving Systemic Cancer Therapy: ASCO Guideline Questions and Answers. JCO Oncol Pract. (2023) 19:718–23. doi: 10.1200/op.23.00263, PMID: 37459585

[49] Larocca A Bonello F Gaidano G D’Agostino M Offidani M Cascavilla N . Dose/schedule-adjusted Rd-R vs continuous Rd for elderly, intermediate-fit patients with newly diagnosed multiple myeloma. Blood. (2021) 137:3027–36. doi: 10.1182/blood.2020009507, PMID: 33739404

